# The ICR1000 UK exome series: a resource of gene variation in an outbred population

**DOI:** 10.12688/f1000research.7049.1

**Published:** 2015-09-22

**Authors:** Elise Ruark, Márton Münz, Anthony Renwick, Matthew Clarke, Emma Ramsay, Sandra Hanks, Shazia Mahamdallie, Anna Elliott, Sheila Seal, Ann Strydom, Lunter Gerton, Nazneen Rahman

**Affiliations:** 1 Division of Genetics & Epidemiology, The Institute of Cancer Research, London, SM2 5NG, UK; 2 Wellcome Trust Centre for Human Genetics, Oxford, OX3 7BN, UK; 3 Cancer Genetics Unit, Royal Marsden NHS Foundation Trust, London, SM2 5PT, UK

**Keywords:** exome, exome sequencing, next-generation sequencing, NGS, population genetics, variant, gene variation

## Abstract

To enhance knowledge of gene variation in outbred populations, and to provide a dataset with utility in research and clinical genomics, we performed exome sequencing of 1,000 UK individuals from the general population and applied a high-quality analysis pipeline that includes high sensitivity and specificity for indel detection. Each UK individual has, on average, 21,978 gene variants including 160 rare (0.1%) variants not present in any other individual in the series. These data provide a baseline expectation for gene variation in an outbred population. Summary data of all 295,391 variants we detected are included here and the individual exome sequences are available from the European Genome-phenome Archive as the ICR1000 UK exome series. Furthermore, samples and other phenotype and experimental data for these individuals are obtainable through application to the 1958 Birth Cohort committee.

## Introduction

The advent of exome sequencing has increased our ability to comprehensively capture gene variation, furthering both discovery of genes predisposing to disease and the expansion of clinical genomic sequencing. In both contexts the spectrum and frequency of gene variation in the general population is a necessary consideration when evaluating potential association with disease. Although publicly accessible summary exome datasets such as the Exome Sequencing Project and Exome Aggregation Cohort (ExAC) series are available, these are compilations of exome data from disease cohorts rather than the general population, and individual level data is not provided, hampering utility ^1, 2^. The 1000 Genomes project has made individual population-based exome data available. However, the sample sizes for any given population range from 61 to 113 individuals, limiting detection of rarer variation ^3^. Some larger population datasets are available; studies of 2,636 Icelanders and of 250 Dutch individuals have been recently published ^4, 5^, although individual sequences are not accessible, impeding direct comparative analyses of data from different populations. Moreover the spectrum of variation from founder populations, such as the Icelanders, is not representative of outbred populations, particularly in relation to rare variation. This is of crucial importance in clinical genomics because the majority of disease-causing gene variants are very rare.

A further limitation of some of the available datasets is the sub-optimal detection of insertions and deletions (collectively termed ‘indels’). Many publications of these datasets have either excluded all/some indels, or have explicitly stated that the indel calling is not high-quality ^1, 3, 6– 8^. This has potential to severely compromise clinical genomic utility, as indels are a major class of pathogenic variant, and are routinely and robustly detected by pre-NGS mutation detection methods, such as Sanger sequencing.

Here we have sought to enhance knowledge of rare gene variation in outbred populations and to provide an exome dataset with utility in research and clinical genomics. To this end we have generated exome data in 1,000 UK individuals from the general population (the ICR1000 UK exome series) and applied an analytical pipeline with high sensitivity and specificity for indel detection.

We included 1,000 samples from the 1958 Birth Cohort, selecting individuals with self-reported white British ancestry. The 1958 Birth Cohort is a population series of individuals born in the UK during one week in 1958. It is a longitudinal study collecting information on numerous aspects of development with the first sweep of data collection in 1965 and the most recent in 2013. Biomedical assessment was undertaken during 2002–2004, at which time blood samples and informed consent were obtained for creation of a genetic resource ( www.cls.ioe.ac.uk). The advantages of this series are: first, it is population-based and unselected with respect to disease. Second, unlike most published exome series we are able to make the individual exome sequences available, greatly enhancing utility. Third, phenotype data, DNA and cell lines from individuals in whom we performed exome sequencing are available to researchers following a successful application to the 1958 Birth Cohort committee.

We performed exome sequencing using the Illumina TruSeq exome and Illumina instruments. Average coverage of the target across all individuals was 47×. Median overall coverage of the target at 15× was 91% across the 1,000 individuals, with a median of 47,240,000 reads mapping to the target. The FASTQ files for all individuals are available from the European Genome-phenome Archive (EGAD00001001021). We have called the dataset the ICR1000 UK exome series.

We analysed the data with the OpEx pipeline (version 1.0.0) which has high sensitivity (95%) and specificity (97%) for indels, with a low false discovery rate (3.4%). OpEx includes Stampy v1.0.14 for alignment, Platypus v0.1.5 for variant detection and CAVA v.1.1.1 for variant annotation ^9– 11^. Of particular note, the default OpEx indel calls are consistent with the clinical convention (i.e. called at the most 3’ position in the coding strand) but if alternative representation(s) are possible this is noted and the call at the most 5’ position is also provided ^11^.

**Figure 1.**
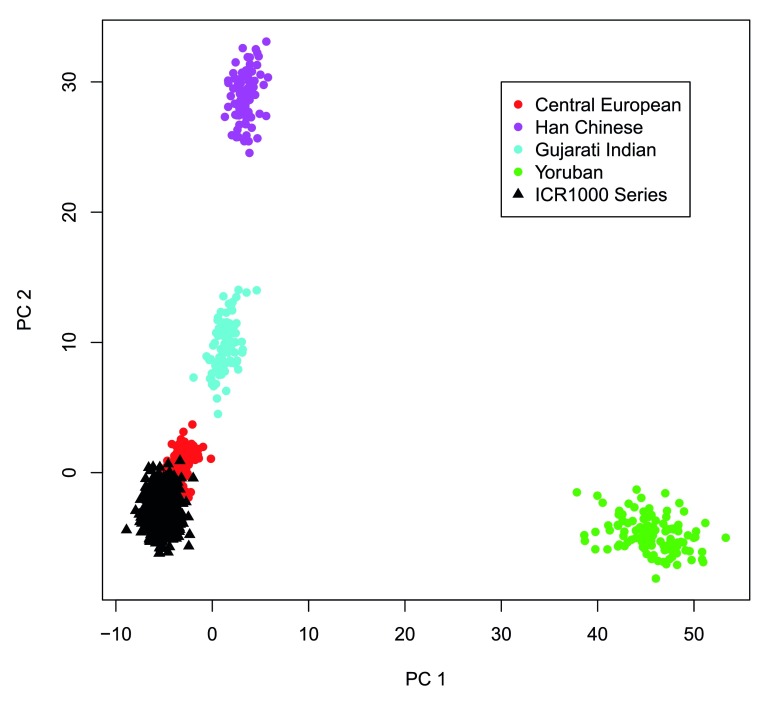
Population structure of the ICR1000 UK exome series. Plot of first and second principal components from PCA using HapMap populations and the ICR1000 UK exome series showing that the series clusters with the Central European population, with no ethnic outliers.

To confirm the series provides representation of individuals with similar ancestry, we performed principal component analysis with the HapMap data for the Central European, Han Chinese, Gujarati Indian, and Yoruban populations ^12^. All individuals clustered tightly with the Central Europeans, with no evidence of ethnic outliers ( Figure 1).

**Figure 2.**
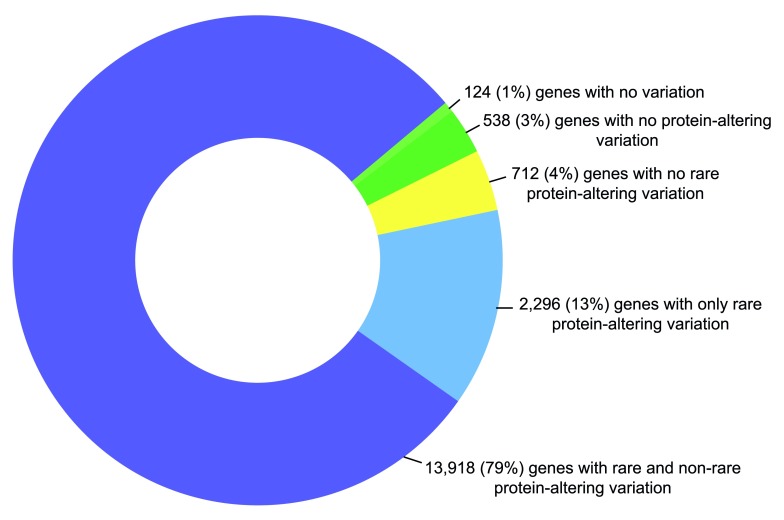
Summary of gene variation in the ICR1000 UK exome series. Protein-altering variation includes nonsynonymous variants, inframe indels and protein-truncating variants (i.e. frameshifting indels or variants that alter essential splice-site residues). The majority of genes include variants across the frequency spectrum.

**Supplementary Figure 1.**
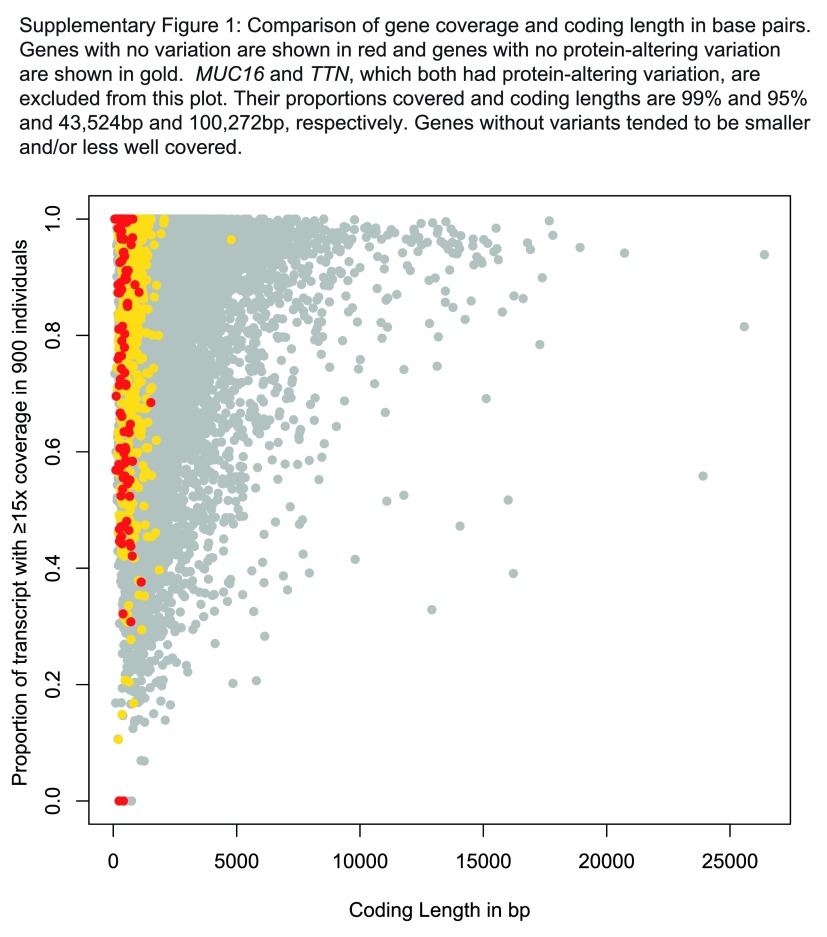
Coverage and coding length of genes. Scatterplot of gene coverage in 90% of samples vs the coding length in base pairs.

We evaluated 19,922 genes from the Ensembl database, of which 17,588 passed our sequence performance criteria and were included in the analyses outlined below ( Supplementary Table 1). We calculated the percentage of coding bases that achieved coverage of at least 15×, the same threshold at which variant calling was optimised. This allows one to place a gene’s observed variation in the context of what was successfully sequenced, an essential requirement in clinical genomics. The genes included and the percentage successfully covered is given in Supplementary Table 1. We observed variation in 17,464 of the 17,588 genes ( Figure 2). The 124 genes with no variation had less than 1.1kb of coding sequence and/or less than 80% of coding sequence successfully sequenced at 15× ( Supplementary Figure 1). The 538 genes with no protein-altering variation were similarly enriched for smaller genes ( Supplementary Figure 1). 712 genes had no rare (0.1%) protein-altering variants. Conversely, 2,296 genes had only rare protein-altering variants. The remaining genes had variants across the frequency spectrum ( Figure 2, Supplementary Table 2).

**Figure 3.**
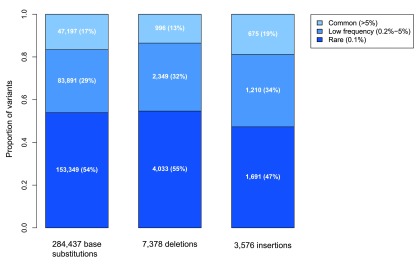
Summary of variants in the ICR1000 UK exome series by type and frequency. The number and percentage of variants in each category is shown in white text. For all variant types, rare variants predominate, and the distribution of variants of different frequencies is similar.

We outputted all variants in exons or within 10 bp of the intron-exon boundary and detected 295,391 distinct variants in total: 284,437 base substitutions, 7,378 deletions and 3,576 insertions ( Figure 3). Summary information for all 295,391 variants is given in Supplementary Table 2. We stratified the variants by frequency and type. We term variants present in only one individual i.e. with frequency of 0.1%, ‘rare’, those with frequency between 0.2–5% (i.e. present in 2–50 individuals) ‘low-frequency’ and those with frequency >5% (i.e. present in >50 individuals) ‘common’. The majority of the 295,391 variants were rare (54%, n=159,073), with 30% (n=87,450) occurring at low frequency and 16% (n=48,868) being common ( Figure 3, Supplementary Table 2).

We first considered the quality of the base substitution calls. The overall transition/transversion (Ti/Tv) ratio was 3.05, confirming our low false detection rate. The Ti/Tv ratio increased from 3.17 to 3.25 as frequency decreased from common to low as anticipated ^13^. The Ti/Tv ratio of the rare variants was lower, as expected, at 2.91. This is because rare variants are enriched for nonsynonymous variants, a class more likely to contain transversions, thus resulting in a lower Ti/Tv ratio ^4, 8^. The nonsynonymous/synonymous ratios were 0.90, 1.46, and 1.86 for the common, low, and rare base substitutions respectively, showing good agreement with previous estimates ^3^. In total we identified 284,437 base substitutions; 99,568 synonymous, 158,932 nonsynonymous, 22,613 in flanking intronic sequence and 3,324 affecting stop codons (3,210 stop-gain, 114 stop-loss). 54% (n=153,349) of the base substitutions were rare, 29% (n=83,891) were low frequency and 17% (n=47,197) were common ( Figure 3, Supplementary Table 2).

**Figure 4.**
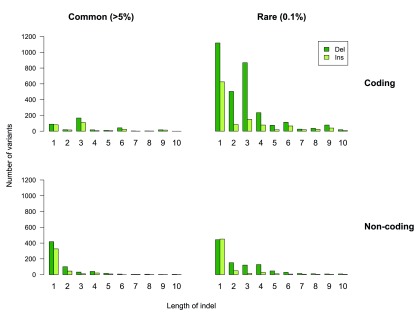
Summary of indel characteristics in the ICR1000 UK exome series. Variant frequency varies with type and length of indel. Deletions are more common than insertions, particularly for rare variants. There is enrichment of indels of 3 bp, 6 bp and 9 bp in coding but not non-coding sequence, because these cause inframe variants.

We next considered the insertions and deletions. We identified 10,954 indels in total, of which 52% (n=5,724) were rare, 33% (n=3,559) were low frequency and 15% (n=1,671) were common ( Figure 3, Supplementary Table 2). The type and length of indel detected influenced variant frequency and we detected twice as many deletions as insertions ( *P*=8.68×10 ^-289^), in line with previous data ( Figure 4) ^4, 14^. For coding indels the majority of common variants were inframe 3 bp variants or 1 bp deletions or insertions. However, we observed a different distribution for rare coding indels, with more frameshifting single base indels than inframe 3 bp indels ( *P*=1.2×10 ^-10^; Figure 4). This likely reflects selection against frameshifting indels becoming common, as they are more often biologically deleterious than inframe mutations.

Our data allow us to describe the average spectrum of gene variation of a UK individual born in 1958, who has 21,978 gene variants (range=19,637–23,009). This includes 9,993 synonymous variants (range=8,921–10,406), 8,718 nonsynonymous variants (range 7,834–9,114), 418 deletions (range=290–476) and 289 insertions (range 234–333) ( Table 1, Supplementary Table 3).

**Table 1.** Average number of exome variants per UK individual.

	Total	Common >5%	Low Frequency 0.2–5%	Rare 0.1%
All Variants	21,978	20,970	848	160
**Stratified by sequence change**				
Base substitution	21,270	20,302	814	154
Deletion	418	392	22	4
Insertion	289	276	12	2
**Stratified by functional impact**				
Synonymous (SY)	9,993	9,632	313	48
Nonsynonymous (NSY)	8,718	8,221	407	89
Splice-site (SS, SS5)	2,494	2,396	86	12
Exon end (EE)	396	375	17	4
Inframe (IF)	147	137	8	1
Frameshifting indel (FS)	99	90	6	3
Stop-gain (SG)	57	49	5	2
Essential splice-site (ESS)	55	50	3	1
Initiating methionine (IM)	10	10	1	0
Stop-loss (SL)	10	9	0	0

The ranges are given in Supplementary Table 3. Values are rounded to the nearest whole number. The functional impact class supplied by CAVA is given in parentheses. Full details of the CAVA classification system are given in Münz *et al*. ^11^

We believe these results and the underlying raw ICR1000 UK exome data have considerable utility in scientific and translational research and in clinical genomics.

## Methods

### Samples

We used lymphocyte DNA from 1,000 individuals with self-reported white British ancestry obtained from the 1958 Birth Cohort Collection, a continuing follow-up of persons born in the United Kingdom in one week in 1958. Biomedical assessment was undertaken during 2002–2004 at which blood samples and informed consent were obtained for creation of a genetic resource ( www.cls.ioe.ac.uk).

### Exome sequencing

We prepared DNA libraries from 1.5 µg genomic DNA using the Illumina TruSeq sample preparation kit. DNA was fragmented using Covaris technology and the libraries were prepared without gel size selection. We performed target enrichment in pools of six libraries (500 ng each) using the Illumina TruSeq Exome Enrichment kit. The captured DNA libraries were PCR amplified using the supplied paired-end PCR primers. Further details are given at http://images.illumina.com/documents/products/datasheets/datasheet_truseq_exome_enrichment_kit.pdf. Sequencing was performed with an Illumina HiSeq2000 (SBS Kit v3, one pool per lane) generating 2×101 bp reads.

### Exome analysis with OpEx

We analysed sequencing reads with the OpEx pipeline. OpEx includes Stampy v1.0.14 for alignment, Platypus v0.1.5 for variant detection and CAVA v.1.1.1 for variant annotation ^9– 11^. The OpEx pipeline is available at http://www.icr.ac.uk/OpEx. Full details of the OpEx description and performance are being prepared for publication. In brief, we validated the OpEx pipeline using 142 independent samples for which we had previously generated extensive data from genotyping and sequencing studies. We evaluated 12,656 sites which included 11,926 common base substitutions with minor allele frequency (MAF) ≥5%, 409 variants validated by Sanger sequencing, and 321 sites known to be negative through variant caller comparison analyses. For base substitutions the sensitivity was 99.7% (12017/12049), specificity was 96% (44/46) and the FDR 0.02% (2/12019). For indels ≤10 bp, the sensitivity was 95% (256/269), specificity was 97% (266/275) and the FDR 3.4% (9/265). The detection of longer indels (>10 bp) was less robust, with higher false detection rates than for small indels, these were therefore excluded from the variant tables and analyses.

In the variant analyses we included base substitution calls for which at least one call across all samples had a QUAL score >100. For deletions we included calls for which at least one call across all samples had both PASS in the FILTER column and ≥20% of reads included the deletion. For insertions we only included calls that had both PASS in the FILTER column and ≥20% of reads included the insertion. Seven samples showed excess heterozygosity and were excluded from the variant analyses.

### Confirmation of OpEx performance in the ICR1000 UK exome series by Sanger sequencing

We selected 116 gene variants that were detected once in the series to further evaluate variant calling performance. This included 31 base substitutions, 36 deletions, and 49 insertions. The 31 base substitutions, 13 of the deletions, and 2 of the insertions occurred in genes for which we had Sanger sequencing primers available in-house. The remaining 23 deletions and 47 insertions were selected randomly from amongst 96 individuals whose DNA was included on the same plate to aid the laboratory work. For these 70 indels, we generated FASTA sequence for the 500 bp window surrounding the called variant using the BSgenome package in R ^15^. We used BatchPrimer3 to design primers with minimum product size of 200 bp, optimal of 350 bp, maximum of 501 bp, max Tm difference of 5.0, and default for all other settings. We performed PCR reactions using the Qiagen Multiplex PCR kit, and bidirectional sequencing of resulting amplicons using the BigDye terminator cycle sequencing kit and an ABI3730 automated sequencer (ABI PerkinElmer). All sequencing traces were analysed with both automated software (Mutation Surveyor version 3.10, SoftGenetics) and visual inspection.

113 of the 116 calls were detected by Sanger sequencing. Only one of the validated variants had a discrepant annotation; an insertion of 1 bp in a run of T’s in the OpEx call appeared to be a deletion of 1 bp in the Sanger sequence. There were three false positive OpEx calls (FDR = 0.03). Two were insertions of 6 bp and 8 bp present at the end of reads, a common site of false positive calls. The other was a 3 bp deletion in a region with poor mapping quality and poor coverage.

### Transcript selection

We selected transcripts from the Ensembl database (version 65) and evaluated the 19,922 genes present on chromosomes 1–22, X, or Y, that had both a start and stop codon, and were not known pseudogenes as specified in the default exome transcript database supplied with OpEx. The specific transcripts selected are provided in Supplementary Table 1 (see Supplementary Table 4 for descriptions of column headers).

### Coverage evaluation per gene

Gene coverage was evaluated using the coding bases of the selected transcript; intronic and UTR sequence was excluded. 2,334 genes were excluded from the variant analyses because either <50% of coding bases were targeted by the pulldown (n=1,380) or <50% of coding bases were covered at ≥15× in 500+ individuals (n=954). Of the 1,380 genes that were not targeted by the pulldown, 207 had ≥50% of the gene covered at ≥15× in at least one individual and thus represented off-target effects ( Supplementary Table 1).

### Principal component analysis (PCA)

We utilized the genotype data from the 397 unrelated individuals in the Central European, Han Chinese, Gujarati Indian, and Yoruban populations in Phase 3 of the HapMap project ^12^ to perform PCA. The genotypes were evaluated for 2,577 base substitution variants on chromosome 1 which were called in both the exome data and the HapMap data. PCA was performed using the prcomp function in R. To allow confident imputation of reference homozygotes in the exome data, variants were required to have ≥13× coverage at the position for every individual. Ten individuals were excluded from PCA analysis as fewer than 90% of the variants met the coverage requirement. 

### Detection of gene variation

We assessed variation in the 17,588 genes with good coverage. Variants in exons or flanking sequence up to ten bases into the intron were outputted and included in Supplementary Table 2 (see Supplementary Table 4 for descriptions of column headers). Variants detected in only one individual in fewer than 15 reads were excluded. Multi-allelic variants were separated into their constituent alleles for the individual-level and gene-level analyses. For the individual-level analyses, the most severe consequence was selected for variants that affected multiple genes based on the functional impact class supplied by CAVA ^11^. For the gene-level analyses, variants that affected multiple genes were included as variants in each gene. We defined coding indels as those that affected any exonic or essential splice-site (+1, +2, -1, -2) residue, and non-coding indels as those that affected residues +3 to +10 or -3 to -10.

### Statistical analyses

The Ti/Tv and nonsynonymous/synonymous ratios were calculated in R using the exonic base substitutions. The overall proportion of deletions amongst the 10,954 indels (0.67) was tested for significant difference from 0.5 using the prop.test function in R. The number of 1 bp and 3 bp coding deletions was compared between rare and common variants using a 2×2 contingency test with the chisq.test function in R.

### ICR1000 UK exome series availability

The FASTQ files for all 1,000 individuals have been deposited in the European Genome-phenome archive (EGA). The accession number is EGAD00001001021.

The files are available at https://www.ebi.ac.uk/ega/datasets/EGAD00001001021.

Details of how to request access to the data are available at www.icr.ac.uk/ICR1000exomes.

Researchers and authors that use the ICR1000 UK exome series should reference this paper and should include the following acknowledgement: "This study makes use of the ICR1000 UK exome series data generated by Professor Nazneen Rahman’s Team at The Institute of Cancer Research, London”.

## Figures and Tables

**Supplementary Table 1.** Summary information for genes. Transcript, coverage, and variation information for 19,922 genes from the Ensembl database (v65). Click here for additional data file..

**Supplementary Table 2.** Summary information for all variants detected in the ICR1000 UK exome series. Variant information for all 295,391 exome variants detected in the ICR1000 series. Click here for additional data file..

**Supplementary Table 3.** Range and average number of variants in the ICR1000 UK exome series. The range and average number of variants in the ICR1000 series by variant type and frequency. Click here for additional data file..

**Supplementary Table 4.** Column descriptions for supplementary tables. Descriptions of column headers for Supplementary Table 1 and Supplementary Table 2. Click here for additional data file..
