# Supplementary material for: The ICR1000 UK exome series: a resource of gene variation in an outbred population
Source: F1000Res. 2015 Sep 22;4:883. [Version 1] doi: 10.12688/f1000research.7049.1 (PMC4706061; doi:10.12688/f1000research.7049.1)
Supplement: Peer review history

# Peer review: Referee response for version 1

**DOI:** 10.5256/f1000research.7589.r10831

**Published:** 2015-11-27

**Authors:** Klaudia Walter

**Affiliations:** 1 Department of Human Genetics, Wellcome Trust Sanger Institute, Cambridge, UK

The authors performed exome sequencing of 1000 UK individuals from the 1958 Birth Cohort who are representative of an outbred population. This data set will be useful for the research community and for clinical genomics, especially since it provides individual genotypes from high-coverage data with a high-quality INDEL calling pipeline.

The study is very clear and well described and it will provide a good quality data set for further research. 

It would be interesting to compare the variant calls of this study to the population based UK10K-cohorts call set which is based on low-coverage whole-genome sequencing of 3781 samples (UK10K Consortium, 2015).

The authors say low-frequency variants with allele frequencies between 0.2–5% are variants present in 2–50 individuals. It would have been more accurate to refer to allele counts of 4-100 instead, since the allele number is 2000 for 1000 individuals.

I was wondering whether it would have been possible to keep all individuals in the principle components analysis if a different set of variants had been selected for analysis.

In summary, the exome sequencing data set of 1000 UK individuals will provide the research community with a useful resource, a European based set of variants and especially a high- quality set of INDELs.

I have read this submission. I believe that I have an appropriate level of expertise to confirm that it is of an acceptable scientific standard.

# Peer review: Referee response for version 1

**DOI:** 10.5256/f1000research.7589.r10652

**Published:** 2015-10-29

**Authors:** Fiona Cunningham

**Affiliations:** 1 European Molecular Biology Laboratory, Wellcome Trust Genome Campus, European Bioinformatics Institute, Cambridge, UK

The authors describe ICR1000, a UK-based reference data set for variation. Using 1000 consented samples from the 1958 UK birth cohort, they performed deep exome sequencing to a median depth of 15X. The samples were chosen from those who self-reported as having white British ancestry though does not comprise samples from individuals of other ancestry in the general British population. All data have been deposited in the EGA, and so are available as a resource to other bona fide researchers.

This is a thorough and well performed study that includes detailed methods and results. In particular the authors have developed a pipeline with high sensitivity and specificity for detecting indels. This results in findings of nearly 11,000 indels, over half of which are novel and in total over 159,000 rare variants were found (with frequency of 0.1%). It would be helpful if the authors could include a comparison to the UK10K project. This was a major UK-based initiative that sequenced 4000 genomes of individuals with deep phenotype data and these data are also available in the EGA.

In conclusion, this is valuable work that found new rare variants that will improve exome analysis. In particular, identification of novel indels, which have not been well characterised in many other studies, is of importance to the field and will be of benefit to clinical genomics where correct interpretation of rare variants is key.

---

Here is a citation for the database mentioned in the paper. Including this is helpful for continued development and funding. 

European Genome-phenome Archive:

Lappalainen, I., Almeida-King, J., Kumanduri, V., Senf, A., Spalding, J.D., ur-Rehman, S., Saunders, G., Kandasamy, J., Caccamo, M., Leinonen, R., *et al.* (2015). The European Genome-phenome Archive of human data consented for biomedical research. Nat Genet 47, 692–695.

I have read this submission. I believe that I have an appropriate level of expertise to confirm that it is of an acceptable scientific standard.

# Peer review: Referee response for version 1

**DOI:** 10.5256/f1000research.7589.r10592

**Published:** 2015-09-30

**Authors:** Gail Jarvik

**Affiliations:** 1 Division of Medical Genetics, University of Washington, Seattle, WA, USA

This is well executed and data are available.  If anything the authors are modest about impact.  My report is below:

Dr Rahman and her colleagues have summarized both variant and in/del variation in 1000 UK Biobank participants and shared these data.  Their analyses show the rate of common and rare variation in the participants. While these estimates are important and underscore that rare variation is very common, possibly the largest impact of this work is the ability to assess the frequency of in/dels in a population-based cohort. Allele frequency data are among the most important consideration in determining whether a variant is pathogenic or not. Clinical laboratories and researchers alike rely on public databases for minor allele frequency data.  As the authors rightfully point out, the ExaC variant database is a compilation of individuals with various diseases, which can skew allele frequencies. The Exome Variant Server is limited to heart, lung, and blood phenotypes, but contains no in/del data. While there is limited data within ancestry groups here, the well-curated in/del data will be a boon to those classifying variant pathogenicity. Further, the ability to access the phased sequence data provided here adds utility.  This is an important resource that adds to our understanding of rare human variation.

I have read this submission. I believe that I have an appropriate level of expertise to confirm that it is of an acceptable scientific standard.
